# Over-Expression of Cysteine Leucine Rich Protein Is Related to SAG Resistance in Clinical Isolates of *Leishmania donovani*

**DOI:** 10.1371/journal.pntd.0003992

**Published:** 2015-08-21

**Authors:** Sanchita Das, Priyanka Shah, Rati Tandon, Narendra Kumar Yadav, Amogh A. Sahasrabuddhe, Shyam Sundar, Mohammad Imran Siddiqi, Anuradha Dube

**Affiliations:** 1 Division of Parasitology, CSIR-Central Drug Research Institute, Lucknow, India; 2 Molecular and Structural Biology, CSIR-Central Drug Research Institute, Lucknow, India; 3 Department of Medicine, Institute of Medical Sciences, Banaras Hindu University, Varanasi, India; US Food and Drug Administration, UNITED STATES

## Abstract

**Background:**

Resistance emergence against antileishmanial drugs, particularly Sodium Antimony Gluconate (SAG) has severely hampered the therapeutic strategy against visceral leishmaniasis, the mechanism of resistance being indistinguishable. Cysteine leucine rich protein (CLrP), was recognized as one of the overexpressed proteins in resistant isolates, as observed in differential proteomics between sensitive and resistant isolates of *L*. *donovani*. The present study deals with the characterization of CLrP and for its possible connection with SAG resistance.

**Methodology and Principal Findings:**

In pursuance of deciphering the role of CLrP in SAG resistance, gene was cloned, over-expressed in *E*. *coli* system and thereafter antibody was raised. The expression profile of CLrP and was found to be over-expressed in SAG resistant clinical isolates of *L*. *donovani* as compared to SAG sensitive ones when investigated by real-time PCR and western blotting. CLrP has been characterized through bioinformatics, immunoblotting and immunolocalization analysis, which reveals its post-translational modification along with its dual existence in the nucleus as well as in the membrane of the parasite. Further investigation using a ChIP assay confirmed its DNA binding potential. Over-expression of CLrP in sensitive isolate of *L*. *donovani* significantly decreased its responsiveness to SAG (SbV and SbIII) and a shift towards the resistant mode was observed. Further, a significant increase in its infectivity in murine macrophages has been observed.

**Conclusion/Significance:**

The study reports the differential expression of CLrP in SAG sensitive and resistant isolates of *L*. *donovani*. Functional intricacy of CLrP increases with dual localization, glycosylation and DNA binding potential of the protein. Further over-expressing CLrP in sensitive isolate of *L*. *donovani* shows significantly decreased sensitivity towards SAG and increased infectivity as well, thus assisting the parasite in securing a safe niche. Results indicates the possible contribution of CLrP to antimonial resistance in *L*. *donovani* by assisting the parasite growth in the macrophages.

## Author Summary

*Leishmania* causes complex of pathologies called Leishmaniasis and among the several forms visceral leishmaniasis is the precarious one as being fatal, if left untreated. Emergence of resistance against several antileishmanials particularly Sodium Antimony Gluconate (SAG) has severely battered the therapeutic strategy against VL and the resistance mechanism is still vague. Thus, to apprehend the underlying mechanism, previously, a differential proteomics of SAG unresponsive versus SAG sensitive isolates of *L*.*donovani* was done wherein overexpression of Cysteine Leucine Rich protein (CLrP), a member of Leucine rich repeat superfamily, was observed. To scrutinize its involvement in the SAG resistance mechanism, which is till date not investigated, the characterization of CLrP was carried out which revealed its post-translational modification along with its dual existence in the nucleus and in the membrane of the parasite. Further investigation using a ChIP assay confirmed its DNA binding potential. Over-expression of CLrP in sensitive isolate of *L*. *donovani* significantly decreased its SAG sensitivity. CLrP overexpressed parasites have increased infectivity. These results point out towards the CLrP’s contribution to antimonial resistance in *L*. *donovani* by facilitating parasites growth through macrophages. Further studies are required to depict CLrP as a potential drug target to strengthen the present arsenal against *L donovani*.

## Introduction

Infection of *Leishmania* has several clinical manifestations as visceral, cutaneous and mucocutaneous forms of leishmaniasis and visceral leishmaniasis (VL), among these, VL is the deadly form in the absence of proper treatment. In India, particularly the states of Bihar, adjoining areas of West Bengal and Jharkhand themselves carry about half the burden of the world’s account of VL [1]. Sodium antimony Gluconate (SAG), having a chemotherapeutic background of 60yrs against VL, is now obsolete in the endemic areas of Bihar due to widespread resistance to antimonials [2]. The emergence of SAG resistance along with the limited availability of safe and cost-effective antileishmanial agents has worsened the situation and raised the chemotherapeutic challenges. Although, there has been a significant advancement in the treatment of VL, the question of resistance still remains unanswered. The resistance phenomenon has been studied mostly in laboratory mutants that differ a lot from field isolates [3,4,5,6]. Earlier some studies based on closely related metal arsenic were carried out to understand the resistance mechanism but was less worthy as it differs from antimony’s working mechanism in several aspects such as in increasing intracellular calcium and not affecting the glutathione level etc. [4]. Other resistance based studies were mostly carried out on the laboratory prepared mutants. While some studies lay emphasis on clinical isolates, but these were based on several biochemical, biophysical and immunological investigations [5,6]. Actual mechanism could be interpreted by exploring clinical isolates on a molecular level and characterizing the differentially regulated proteins in the resistant field isolates [7,8]. Phenotypes, genomic and proteomic level approaches have been applied to investigate the resistance at cellular and molecular level [9,10,11,12]. In order to understand the mechanism at protein level differential proteomics of sodium antimony gluconate (SAG) sensitive and SAG resistant clinical isolates was done wherein several cytosolic as well as membrane proteins were found to be differentially expressed in a SAG resistant strain of *L*.*donovani* [13]. CLrP was spotted as one of the up-regulated protein in the membrane fraction [13]. CLrP is a member of the superfamily of Leucine-rich repeat (LRR) proteins. With the purpose of gaining an in-depth knowledge about CLrP and to understand its association with SAG resistance, CLrP has been characterized and over-expressed in sensitive isolate of *L*. *donovani* (Ld) to analyze its potential to modulate the parasite’s behavior towards SAG.

## Materials and Methods

### Parasites

Clinical isolates were procured from VL patients from Kala–Azar Medical Research centre, Muzaffarpur, Bihar, India. The isolates from patients, who had responded to chemotherapy by SAG were termed as SAG-sensitive (SAG-S), whereas those who remain unresponded to SAG were termed as SAG-resistant (SAG-R). SAG-S and SAG-R isolates used in the present study were 2001 (S1) and 2039 (R1), 1216 (R2), 761 (R3), whereas Dd8 (S2) strain (MHOM/IN/80/DD8) served as reference strain. All the isolates were maintained *in vitro* in RPMI-1640 medium (10%FCS) (Sigma, USA) at 25°C and their virulence have been retained through regular passage in hamsters, so as to maintain their chemosensitivity profiles as described elsewhere[14].

### Expression and purification of recombinant Cysteine leucine rich protein (rLdCLrP)

Genomic DNA of *L*. *donovani* was isolated from 10^8^ cultured promastigotes and subjected to RNase (100μg/ml) treatment [15]. CLrP gene was amplified using Taq DNA polymerase (Sigma Aldrich) lacking 3’-5’ exonuclease activity in a thermocycler (Bio-Rad) under conditions at one cycle of 95°C for 5min, 30 cycles of 95°C for 1min, 60°C for 45s, 72°C for 1.5min, and finally one cycle of 72°C for 10min (Table A in S1 Text). Amplified PCR product was gel eluted from the gel (electrophoresed in agarose gel) by Gen Elute columns (Qiagen). Eluted CLrP was cloned in pTZ57R/T (T/A) cloning vector (Fermentas) and transformed into competent DH5α cells. LdCLrP was further sub cloned at the *Bam*HI and *Eco*RI site of vector pET-28a(+) (Novagen) and transformed in *Escherichia coli* BL21 Strain. The positively transformed cells were inoculated into 5ml test tube culture medium (Luria Bertani) and allowed to grow at 37°C in a shaker at 220 rpm. Cultures in logarithmic phase (at OD_600_ of ~0.5–0.6) were induced for 5hrs with 1mM isopropyl-ß-D-thiogalactopyranoside (IPTG) at 18°C. The lysis of induced as well as uninduced cells was done in SDS- sample buffer (5X stock (0.313M Tris-Hcl (pH6.8), 50% glycerol, 10%SDS) [16]. These SDS-PAGE separated proteins were transferred onto a nitrocellulose membrane as described elsewhere [17]. Blocking of membrane was done using 3% skimmed milk for 1h followed by a 2h incubation with 1:2500 dilution of mouse anti-His antibody (Novagen). This step was followed by incubation with 1:10,000 dilution of goat anti-mouse HRP conjugate antibody (Bangalore Genei) for 1h. All the incubations were performed at room temperature. The blot was developed using an ECL kit (GE Biosciences). For further purification of rLdCLrP, 200ml of LB medium containing 35μg/mL kanamycin was inoculated with *E*. *coli* BL21 strain positively transformed with LdCLrP+pET28a^+^ and grown till O.D._600_ reaches to 0.6 at 37°C. The culture was induced by addition of 1mM (IPTG, Sigma) and then incubated for 8 hrs at 18°C. The rLdCLrP was purified by 6-His Tag fusion peptide derived from the pET28a^+^ vector by affinity chromatography using Ni^2+^ chelating resin. The cells were resuspended in 5mL of lysis buffer [10mM Tris-HCl (pH 8.0), 200mM NaCl] containing 1:200 dilution of the protease cocktail inhibitor (Sigma) with a 30 mins incubation on ice followed by sonication for 10×20s (with 30s interval between each pulse). After sonication the cells were centrifuged at 15,000g for 30min, and the collected supernatant was incubated at 4°C for 1h with the 2ml of Ni-NTA Superflow resin (Qiagen, Hilden, Germany) equilibrated prior with lysis buffer. After washing with buffer (10mM Tris-HCl, 200mM NaCl) containing 10, 20, 30 and 50mM concentrations of imidazole, the purified rLdCLrP was eluted with elution buffer (10mM Tris-HCl, 200mM NaCl, and 300mM imidazole, pH 7.5). Estimation of the protein content in eluted fractions was carried out by the Bradford method and were analysed in 12% SDS-PAGE.

### Molecular modeling

For protein homology analysis and structure prediction, Hhpred (toolkit.tuebingen.mpg.de/hhpred) was used. HHpred constructs a Hidden Markov Model based profile using query sequences against known databases to search for closest possible homolog with HMM profile-profile comparison. PDB database was chosen here for profile analysis and homolog searching. Top scoring PDB was selected as template for further model building. Structure of query sequence was constructed using Modeller9.11 [18] interfaced with Hhpred server based on template 1z7x. Molecular modeling revealed that each conserved fragment consists of a short beta strand and helical coil. Motif analysis was done with ClustalW [19]. Prediction with TMpred [20] programs indicated two transmembrane regions (residues 47 to 70 and 160 to 183) might be present in the protein. However HMMTOP [21, 22] and TMHMM (www.cbs.dtu.dk/services/TMHMM/) servers predicted no transmembrane domains in the amino acid sequence of *L*. *donovani* LLR.

### Phylogenetic analysis

PSI-BLAST was implied to search for potential sequence homologues searches against the nr database for 10 iterations. The E-value cutoff was set to 10–5. This allows detection of far-off members of the sequence family that simple pair wise comparison would fail to disclose. A sequence alignment was generated using alignment program MUSCLE [23]. Sequences with less than 90% identity with each other were selected only and mapped to the uniprot id (HH filter http://toolkit.tuebingen.mpg.de/hhfilter). Phylogenetic tree was calculated using phylogeny.fr (phylogeny.lirmm.fr) with default values of parameters (Fig A in S1 Text).

### Soluble and membrane fraction sample preparation

The preparation of soluble *L*. *donovani* promastigote antigen (SLD) has been described elsewhere [24]. Briefly, mid-phase promastigote (10^9^) (3 to 4 days old culture) were washed 4 times in cold 1×PBS, further resuspended in 1×PBS with protease inhibitor mixture (Sigma-Aldrich), and further ultrasonicated and centrifuged at 40,000g for 30 min. The quantification of protein content of the supernatant was done and the sample was prepared in SDS PAGE sample buffer [25].

### Raising of polyclonal antibodies against rLdCLrP and western blot analysis

Polyclonal antibodies of rLdCLrP have been raised in rabbit (New Zealand white rabbit) with the purified recombinant CLrP as described elsewhere [15]. For Western blot analysis, purified rLdCLrP and whole cell lysate (WCL) (*Leishmania* as well as *E*. *coli* induced and uninduced) was resolved in 12% SDS-PAGE and transferred onto nitrocellulose membrane [17]. The membrane was incubated with antiserum to rLdCLrP (raised in rabbit) at a dilution of 1:5000 for 2 hrs at room temperature (RT) after 2 hrs blocking in 5% BSA. After washing with PBS containing 0.5% Tween 20 (PBS-T) (1×) the membrane was incubated with Rat anti-rabbit IgG HRP conjugate (Invitrogen, Carlsbad, USA) for 1h at RT at a dilution of 1:10,000. Blot has been developed by using diaminobenzidine+imidazole+H_2_O_2_ (Sigma).

### Immunolocalization of CLrP

Cellular localizations of CLrP have been verified by immunofluorescence using anti-rLdCLrP antibody raised in rabbit. *L*. *donovani* parasites (S1) were plated on 18mm cover-slips prior coated with poly-L lysine. These samples were fixed using 4% paraformaldehyde. 0.5% Triton X-100 has been used to permeabilized two of them followed by 1×PBS washing. Both the permeabilized as well as one non-permeabilized cover-slips were incubated with anti-CLrP. These were further treated with secondary anti-rabbit FITC-conjugate (Bangalore Genei) after washing with 1×PBS. Rhodamine-Concanavalin A (Vector Labs) has been used to treat non-permeabilized samples followed by anti-rabbit FITC-conjugate for 1h at RT (1×PBS washing after each incubation). Cover-slips, mounted upside down on glass slides with Fluorescent Mounting Media (CALBIOCHEM), were visualized under a fluorescence microscope (Eclipse 80i Nikon) using 100X oil objective (1.4 NA). Cells transfected with rLdCLrP+pXG-‘GFP+ were also observed directly under the same fluorescent microscope [26].

### Chromatin Immunoprecipitation (ChIP) assay

To analyze propinquity of DNA with CLrP, a ChIP assay was done as described by with slight modifications [27, 28]. *L*. *donovani* (S1) was grown to log phase (3×10^7^cells/ml) (~40ml) and were fixed for 5min with 1% formaldehyde at 25°C. 2.5ml of 2.5M glycine was added to stop the fixation process, and was further incubated for 5min at 25°C and then washed with 1×PBS. Cells were further resuspended in 2ml ChIP lysis buffer {50mM Tris-HCl (pH8.0), 150mM NaCl, 1% Triton X-100, 0.1% Sodium deoxycholate and protease inhibitor cocktail (Sigma)}. Cells were subjected to sonication (QSONICA Misonix) with 10s pulse at 10% amplitude followed by a 1min pause after each pulse. Sheared cells were visualized under microscope and then pelleted at 12000×g for 10min at 4°C. Protein quantification of the supernatant (chromatin) has been done and 1.0mg protein was used for each ChIP reaction assay. To analyze the DNA proximity with CLrP, cross linked chromatin prepared was subjected to ChIP using anti-rLdCLrP and anti-actin (positive control) [29]. Experiments were performed using pre-immune serum and an irrelevant antibody GRP78 (a kind gift from Dr. E. Handman) as a negative control. Reaction mixtures were incubated for 2h at 4°C with shaking. 5mg Protein-A Sepharose beads was added to this after their pre-blocking with 10μg/ml salmon sperm DNA and 1% (w/v) in ChIP lysis buffer for 2h at 25°C followed by another incubation for 2h at 4°C (with shaking). Washing of beads was done twice with ChIP lysis buffer, high salt lysis buffer (same as lysis buffer, but also containing 500mM NaCl), and then with Tris-EDTA (10mM Tris-HCl, pH8.0, 1mM EDTA). The elution of ChIP complexes were done by adding 200μl ChIP elution buffer (50mM Tris-HCl, pH 8.0, 1% SDS, 10mM EDTA) for two times. The combined supernatants were incubated for 5h at 65°C with 16μ of NaCl (5M) to reverse-crosslink DNA and protein components. The resultant DNA has been precipitated overnight at -20°C with three times volume of absolute ethanol. Pellet obtained after centrifugation at 12000×g at 4°C for 30 min, was further resuspended in 100μl Tris-EDTA, 11 μl of 10X proteinase K buffer and 1μl proteinase K from 20mg/ml stock (MBI Fermentas), followed by incubation at 55°C for 1h. DNA was recovered by silica-KI method, and analyzed by PCR for two different genes. For each PCR reaction, 1 μl purified ChIP DNA was used as the template, whereas for control PCR, DNA was isolated from 10% reverse cross linked input chromatin of actin (positive control) and GRP-78, pre-immune serum (negative control).

### Deglycosylation of LdCLrP

To check post translational modification in LdCLrP, deglycosylation of whole cell lysate of *L*. *donovani* has been carried out with deglycosylation mix (New England Biolabs) as per the manufacturer’s denaturing protocol, followed by western analysis through anti-CLrP antibody.

### Quantitative real time (qRT) and western analysis of CLrP expression in clinical isolates

RNA of log phase promastigotes (S1, S2, R1, R2, R3) (10^7^ parasites) was isolated using Tri reagent (Sigma, Aldrich) followed by DNase treatment and quantified. cDNA was synthesized using First-strand cDNA synthesis kit (Fermentas). qRT-PCR was carried out with 12.5μl of SYBR green PCR master mix (TAKARA), 1μg of cDNA, and 200nM primer (Table A in S1 Text) in a final volume of 25μl. qRT was performed with the following conditions: initial denaturation at 95°C for 10min x 40 cycles, each consisting of denaturation at 95°C for 1 min, annealing at 52°C for 1 min and extension at 70°C for 1min followed by 80°C for 10sec. A melt curve of 87 cycles was set at 52°C for 10sec. Quantifications were normalized to the Ld-actin gene. A no-template control cDNA was included to eliminate contaminations or non-specific reactions. The comparative CT method was used to calculate differences in gene expression [30]. Results are expressed as the degrees of difference between ΔCT values of test and comparator sample (S1) to get ΔΔCT [31]. The normalized expression ratio was calculated as 2^- ΔΔCT^ [32]. The expression profile of protein was analyzed in the whole cell lysate of *L*. *donovani* and fold expression was calculated considering the densitometric values of bands at ~150kDa, ~68kDa, ~41kDa and ~27kDa. The graph was plotted with the fold expression of densitometric values normalized with Ld-actin (loading control).

### LdCLrP overexpression in sensitive isolate (S1) of Ld

LdCLrP gene was PCR amplified from LdCLrP+pET28a(+) construct (Table A in S1 Text) and the product was cloned into *Leishmania* expression vector pXG-‘GFP+ at *Bam*HI and *Eco*RV site [33]. S1 promastigotes of late log phase were washed with transfection buffer (21mM HEPES,pH7.5, 137mM NaCl, 5mM KCl, 0.7mM Na_2_HPO_4_, 6mM glucose). Transfection of the parasites with 20μg of LdCLrP+ pXG-‘GFP+ and pXG-‘GFP+ alone, was performed in a Gene Pulsar (Bio-Rad). The transfectants were allowed for 24h recovery and then were selected with G418 at 5, 10, 20, 50μg/mL [33]. The expression outline of the protein in S1(CLrP+pXG-‘GFP+) (T) and vector control i.e. S1(pXG-‘GFP+) (VC) was observed by the fold expression when maintained at 0μg/mL (T_0_ & VC_0_), 20μg/mL (T_20_ & VC_20_) and 50μg/mL (T_50_ & VC_50_) of G418. Fold expression in different clinical isolates as compared to S1(WT) was done by the densitometric study through chemidoc software (BIORAD). The densitometric values were normalized with Ld-Actin (loading control) [29].

### Growth profile of S1 (LdCLrP+pXG-‘GFP+) and S1 (pXG-‘GFP+)

2×10^6^ promastigotes of both the transfectants were seeded in culture flask and each were counted for 8 days in Neubauer’s chamber. Growth curve was obtained as number of parasites versus days.

### Macrophage infection

To check the role of membrane localization of CLrP in macrophage infectivity, log phase *L*.*donovani* promastigotes of wild type (WT) as well as transfectants (VC and T) (5×10^5^ cells each) were pelleted down and incubated with anti-CLrP and pre immune serum (PIS) for 30 mins and then added to well plates containing 10^4^ J774 macrophages [34]. After 4hrs free parasites were removed and allowed to grow for 24 hrs, the cells were washed two times in 1×PBS, fixed in 100% methanol followed by Geimsa staining. At least 100 macrophages were counted per well for calculating % infected macrophages. The results were plotted as the number of parasites per 100 macrophages.

### 50% inhibitory concentrations (IC_50_s) of transfectants for SAG

IC_50s_ values for Sb(III) and Sb(V) of VC and T maintained at 0μg (VC_0,_ T_0_), 20μg (VC_20_ & T_20_) and 50μg (VC_50_ & T_50_) of G418 were determined in the same manner as described elsewhere [35].

### Statistical analysis

Log [inhibitor] vs. response- variable slope of log dose/response data on the drug has been used to get non linear regression for 50% inhibitory concentrations (IC_50_s) of Sb(V) and Sb(III) [36]. Further one way ANOVA test and a post Tukey test were applied for statistically analyzing the data and are presented as means and standard deviations (SDs) of three determinations from three separate experiments. P value of less than 0.05 was considered significant.

### Ethics statement

The study was approved by the Ethics Committee of the Kala-azar Medical Research Centre, Muzaffarpur, India (Protocol # EC-KAMRC/Vaccine/VL/2007-01) with the prior consent of the human subjects and Institutional Animal Ethics Committee (IAEC) of CDRI for conducting the experiments on the animals (25/08/Para/ IAEC dated 03.08.2011). The protocol and the guidelines of IAEC were bound to the National Guideline of CPCSEA (Committee for the purpose of Control and Supervision on Experiments on Animals) under the Ministry of Environment and Forest, Government of India.

## Results

### Molecular characterization of LdCLrp

**Fig 1:**
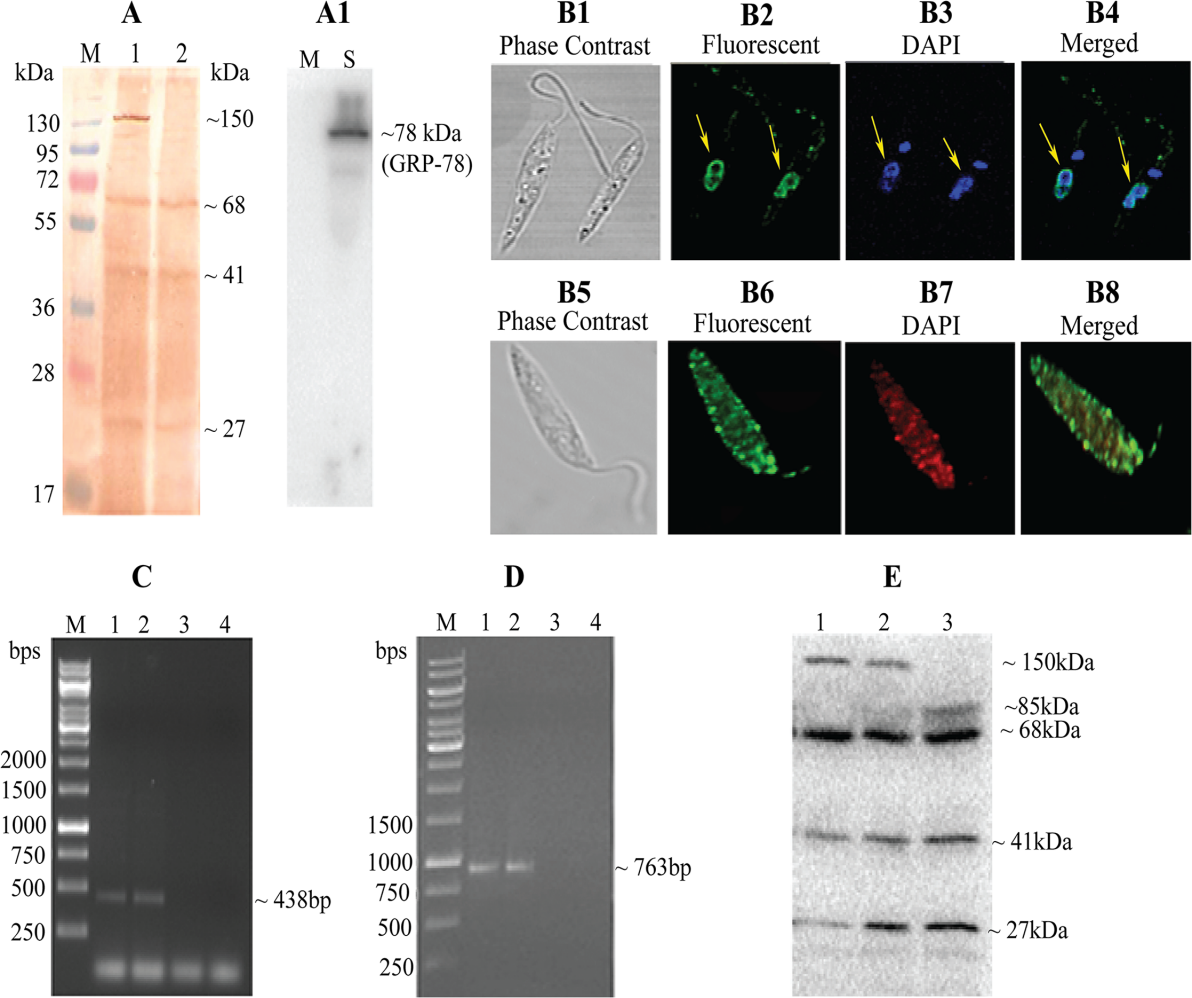
Molecular characterization of *L*. *donovani* CLrP. (A) Western analysis of membrane and soluble fraction of *L*.*donovani* with anti-CLrP antibody Lane1:Molecular mass marker, Lane2: Ld Membrane fraction, Lane3: Ld Soluble fraction, (A1) Lane M: Membrane fraction analyzed with anti-GRP78 antibody, Lane S: Soluble fraction analyzed with anti-GRP78 antibody (B) Immunolocalization of LdCLrP in Ld. B1-B4: Images of permeablized Ld with anti rLdCLrP and then FITC conjugated secondary antibody (arrows shows the nucleus localization of CLrP), (B5-B8) Images of non-permeabilized Ld with anti-rLdCLrP (B5) Phase contrast image (B6) Fluorescent image (rabbit raised anti-CLrP + FITC conjugated rabbit sec antibody) (B7) Fluorescent image (Rhodamine tagged Con A) and (B8) mearged image of B6 and B7. (C-D) ChIP assay probed with Ld genes through PCR (C) 60sRL23a gene (D) TPI gene M:1kb DNA ladder, Lane 1: Chromatin material obtained after immune-precipitation with anti rCLrP, Lane 2: Chromatin material obtained after immune-precipitation with anti-actin, Lane 3: Chromatin material obtained after immune-precipitation with pre-immune serum, Lane 4: Chromatin material obtained after immune-precipitation with anti GRP78. (E) Deglycosylation of WCL of Ld, Lane1: WCL of Ld Lane 2: control, Lane3: Ld+deglycosylase mix.

1866bp CLrP gene has been cloned into T/A vector. The cloned sequence [http://www.ncbi.nlm.nih.gov/nuccore/JQ653307.1 (Accession no. JQ653307.1)] has a close identity of 99.19% to *L*.*infantum*, 95.18% to *L*. *major* and 92.04% to *L*. *mexicana* (Table B in S1 Text). The motif LX3LX2L/CX2LX2LXLX2CX2L is found to be well conserved in the *L*. *donovani* homologue (Fig B in S1 Text). Similar LRR repeat motif was found in amino acid sequence of protein for the homologue of this protein in *L*.*infantum* encoded by LinJ34.0570 gene along with other LRR repeats found in another organism including human, Arabidopsis and yeast (Fig A and B in S1 Text) [37, 38]. For expression and purification of rCLrP further sub-cloning in bacterial expression vector pET28a+ was done, protein was purified and eluted at 300 mM imidazole concentration. The eluted rCLrP was ~71kDa in size as affirmed by the western blot with antibody raised in rabbit (Fig C in S1 Text). Western blot analysis of SLD as well as membrane fraction of *L*.*donovani* promastigote with the polyclonal anti-rLdCLrP antibody detected band of ~68kDa, ~41kDa and ~27kDa protein in the SLD wherein the membrane fraction an additional band of ~150kDa was detected (Fig 1A). To assess the purity of the SLD and membrane fraction western of both fractions was further analyzed with anti-GRP78 antibody (Fig 1A_1_). Dual localization of the protein in the nucleus (Fig 1B1–1B4) as well as in the membrane of the parasite (Fig 1B5–1B8) has been detected in immunolocalization study of CLrP. The nuclear localized CLrP was checked for its association with chromatin material with ChIP assay. The Eluted DNA of ChIP assay was probed with PCR of known genes of *L*. *donovani* i.e. Ld60sRL23a (60s Ribosomal L23a, 438bp) and LdTPI (Triose Phosphate Isomerase, 763bp) (Fig 1C and 1D). Deglycosylation of whole cell lysate of S1 resulted in a band shift of 150kDa band confirming the glycosylation of CLrP at the membrane entity (Fig 1E).

### Expression analysis of CLrP in different clinical isolates

**Fig 2:**
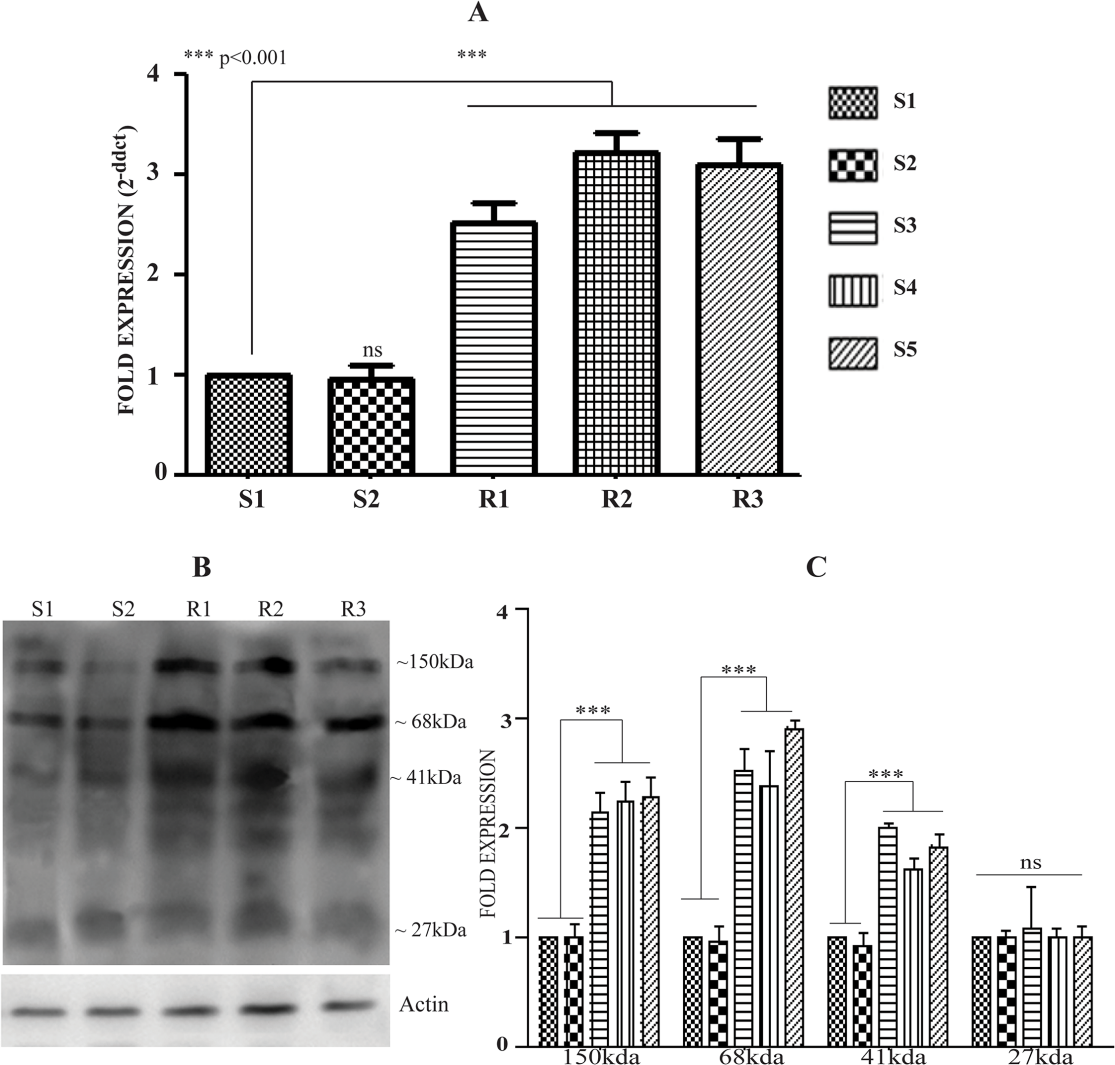
Differential expression of LdCLrP in clinical isolates. (A) Expression analysis of CLrP in different clinical isolates by qRT-PCR. The data are presented as the mean ± SD of three independent RNA preparations. (Asterisks denote highly significant differences from S1) (B) Differential expression pattern of CLrP in different isolates by Western blot of WCL of *L*. *donovani*. Actin served as an internal loading control (C) Fold expression of CLrP in different clinical isolates (densitometric analysis of three different western blots with three independent sample preparations).

The expression pattern of CLrP was investigated in SAG resistant and sensitive strains of *L*. *donovani* through qRT-PCR (Fig 2A). For comparison Ld-actin was employed as internal control which showed a comparable expression level among the clinical isolates (Fig D in S1 Text). The study revealed two fold increased expression of CLrP in all the three resistant isolates as compared to sensitive isolates. The expression level of CLrP was further investigated at protein level (Fig 2B and 2C). In all the resistant strains there was ~2.2 fold increase in the expression of membrane protein (150kDa), wherein the fold expression of 68kDa band increased by ~2.6 fold. 41kDa band was observed to be 1.7 fold higher in resistant isolates. 27kDa band showed no significant difference in its expression among the isolates. Ld-actin was used as loading control.

### Over expression of CLrP in S1

**Fig 3:**
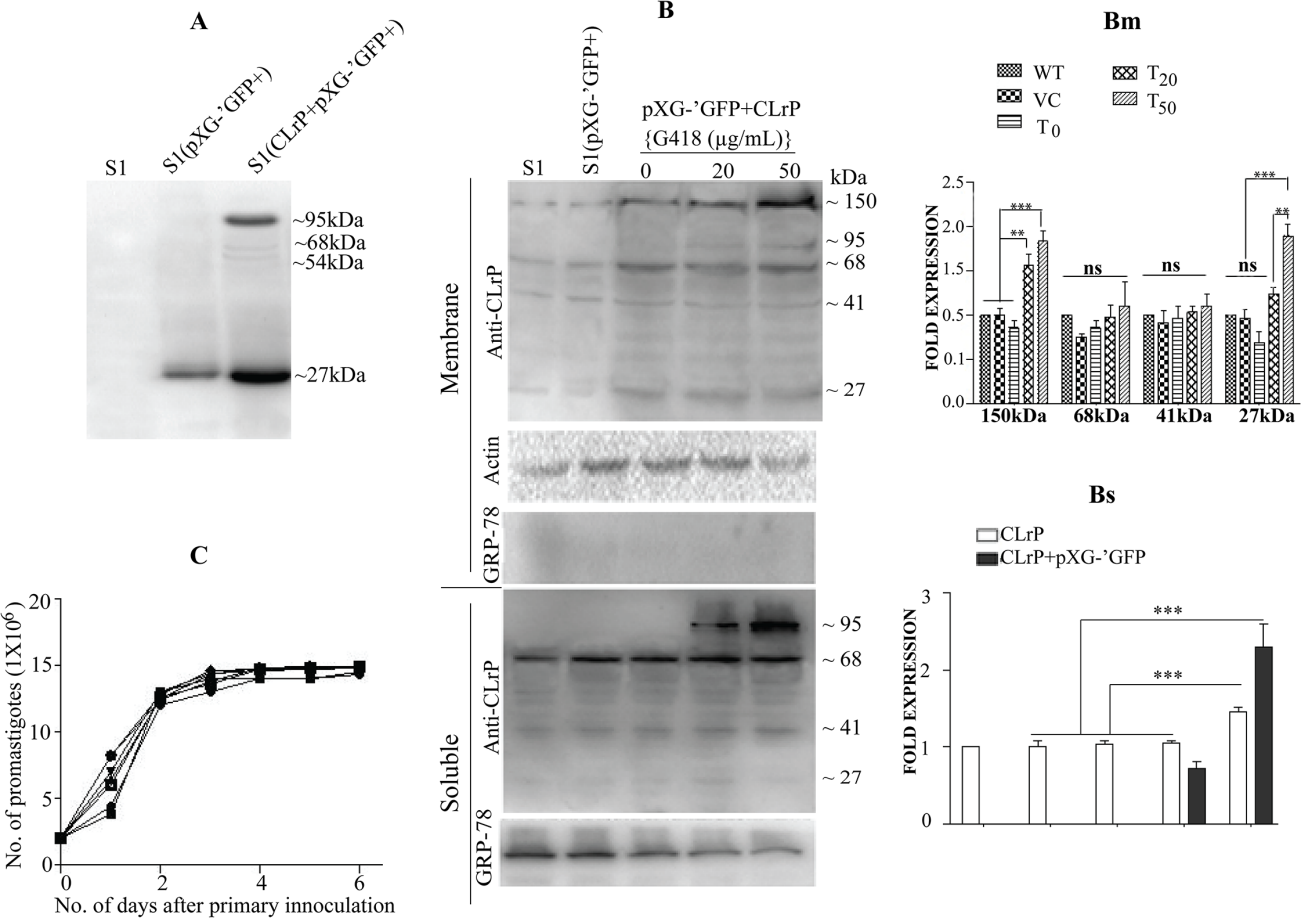
Over expression of CLrP in S1. (A) Western analysis of transfectants with anti GFP. Lane 1 WCL of S1; Lane 2: WCL of VC [S1 (pXG-‘GFP+)]; Lane 3: WCL of T [S1(pXG-‘GFP+LdCLrP)]; (B) western blot analysis of transfectants (membrane and SLD) at various concentrations of G418 using anti rCLrP antibody, anti Actin antibody as an internal loading control, (Bm) Fold expression of CLrP at various concentrations of G418 using densitometric values of three different western blots. [S1 (WT), S1(pXG-‘GFP+) (VC), S1(pXG-‘GFP+CLrP) (T)] (Bs) Expression level of wild type CLrP and fused CLrP+GFP at different concentration of G418 using densitometric values of three different western blots. (C) Growth curve of transfectants (Promastigote).

To assess the role of CLrP in alteration of sensitivity profile of WT, if any, subcloning of CLrP gene was done in pXG-‘GFP+ and then transfected into WT. The immunoblot analysis with WCL of T with anti-GFP antibody exhibited prominent bands at 95kDa and 27kDa, in addition faint bands of ~68kDa and ~54kDa were also evidenced in the same blot which indicates the detachment or parting off of GFP protein from the CLrP+GFP entity after expression. (Fig 3A). VC exhibited a band at mol wt of 27kDa (Fig 3A). The western blot analysis of the soluble as well as the membrane of WT, T and VC parasite (each grown at 0 μg/mL, 20μg/mL and 50 μg/mL of G418) with anti-CLrP revealed the protein expressions with several band patterns (Fig 3B). In the western analysis of membrane fraction with anti-CLrP the 150kDa band was found to be 1.5 fold and 1.8 fold increased with 20 μg/mL (T_20_) and 50 μg/mL (T_50_) of G418 respectively (Fig 3Bm). The intensity of the band (150kDa) remained the same in WT, VC and T growing at 0 μg/mL G418. A similar pattern was observed for 27kDa expression wherein the fold expression increased up to 1.2 to 1.9 fold under 20 and 50 μg/mL G418 pressure respectively. The expression pattern of 68kDa and 41kDa band showed no significant difference under all concentration of G418. The expression pattern of CLrP was again studied in the soluble fraction (Fig 3Bs). There is no significant difference in the 68kDa band expression at 0μg/mL and 20μg/mL of G418, but a significant difference of 1.5 fold existed (parasites grown at 50μg/mL) when compared to the respective band of WT. The fused protein expression appeared at 20μg/mL of G418 in T, with significant difference of 2.2 fold expression when compared to its expression at 50μg/mL G418 (Fig 3Bs). In all transfectants GFP-tagged CLrP was absent without G418 pressure. The growth curve of transfectants [T and VC] were comparable to the WT (Fig 3C).

### Assessment of SAG sensitivity of transfectants *in vitro*

**Fig 4:**
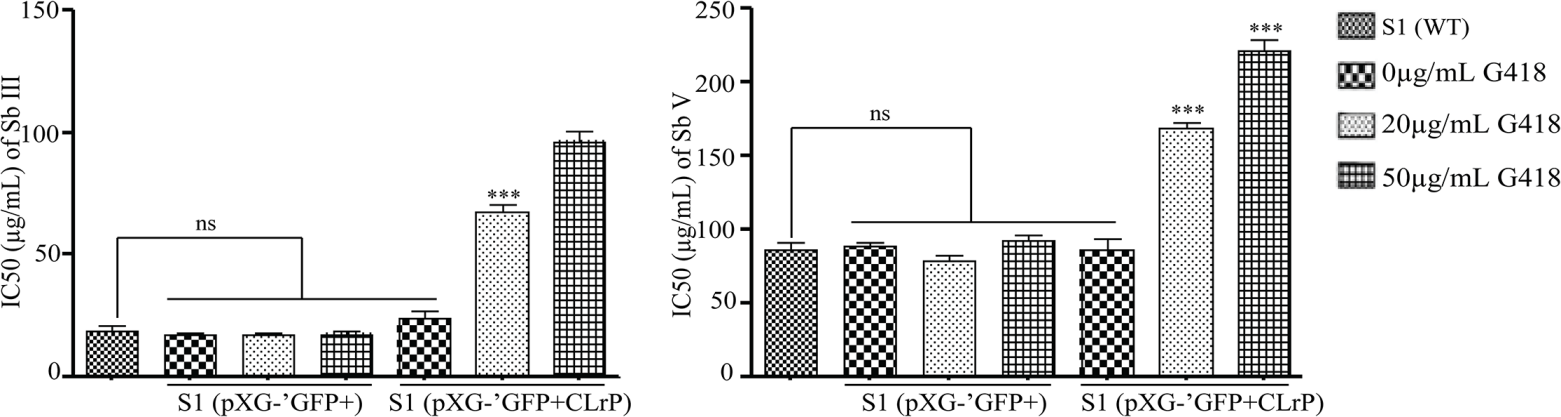
Sensitivity of transfectants to SAG. (A) Sb (III); (B) Sb (V).

Transfectants maintained at 0, 20, 50μg of G418 were assessed for their *in vitro* SAG sensitivity with Sb(V) and Sb(III) in macrophage-amastigote system and promastigotes respectively. Fig 4A and 4B exhibits the sensitivity pattern of the transfectants to Sb(III) and Sb(V). T_50_ has IC_50_ i.e. 221.66±13.14648 for Sb(V) whereas that of VC has 92.50±5.79 as IC_50_.

T_20_ (maintained at 20 μg/mL of G418) also revealed higher IC_50_ (168.60±6.17) as compared to VC_20_(78.55±6.53) whereas T_0_ (growing without G418) exhibited IC_50_ comparable to VC and WT. IC_50_ value for Sb(V) of T_50_ was 2.4 fold amplified than the corresponding VC, whereas for T_20_ it was 1.8 fold increased. There was a similar sensitivity pattern of transfectants to Sb(III) and Sb(V). The sensitivity of T_50_ [IC_50_ = 96.44±7.19] to Sb(III) was found greater than VC_50_ [IC_50_ = 17.67±1.71], whereas at 20μg/mL of G418 it was 67.76±4.82 and VC had similar IC_50_ at all concentrations of G418. T_50_ showed 5.13 fold elevated IC_50_ for Sb (III) as compared to VC_50_. Without G418 the IC_50s_ of transfectants were analogous.

### Infectivity of transfectants

**Fig 5:**
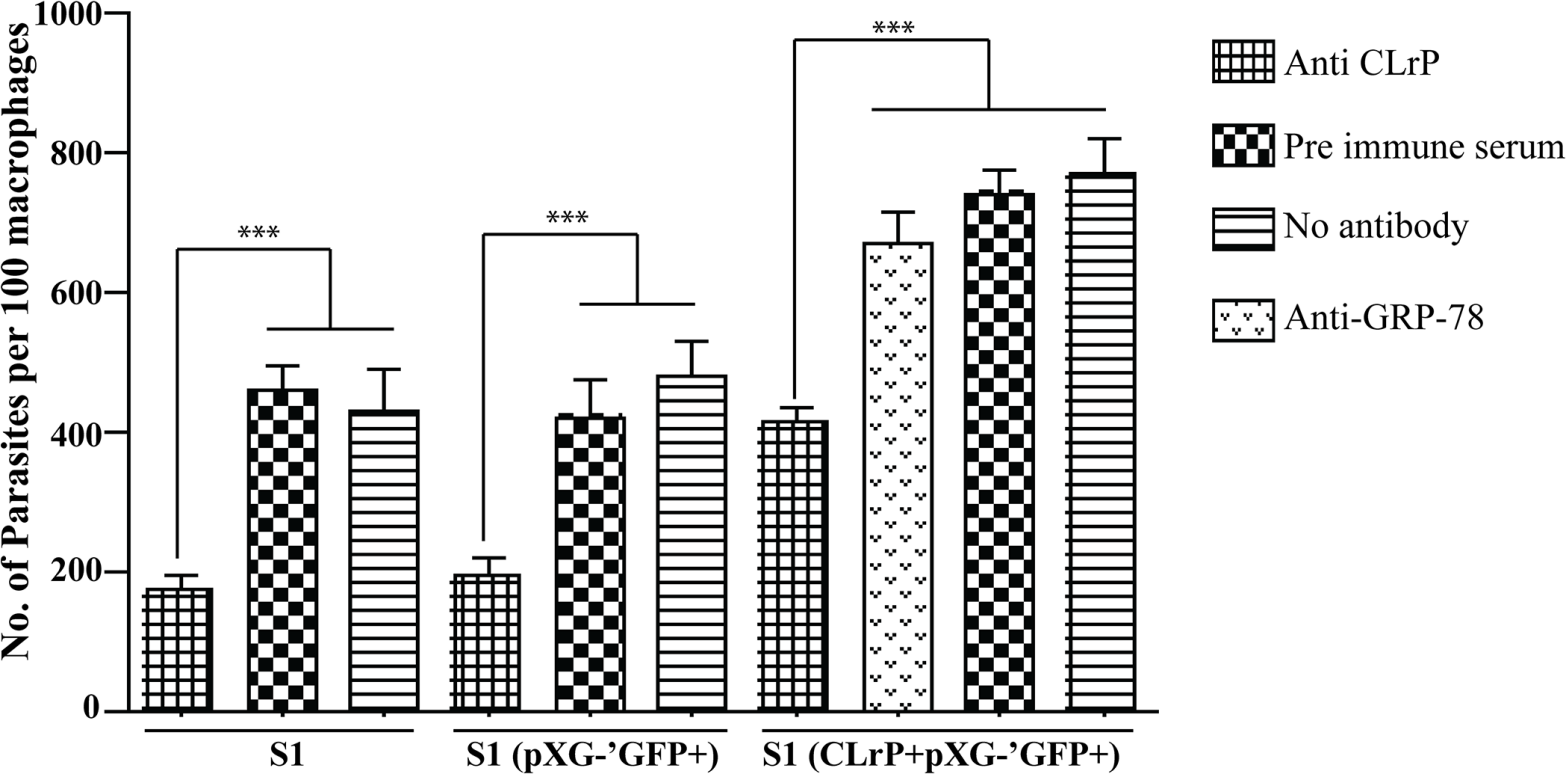
Infectivity assay of anti-rLdCLrP treated promastigotes (wild type as well as transfectants) The data are the ±SD of three independent experiments.

The membrane localization of the protein and glycosylation of CLrP further prompted us to check its infectivity. The infectivity of the macrophage was checked with WT, VC and T parasites blocked with the anti-CLrP antibody, pre immune serum (PIS) was used as a control. Infectivity of S1 and VC was lowered by 2.2 and 2.5 respectively, when compared to control (PIS treated). Parasitic burden (PB) of T was significantly higher than of WT and VC (Fig 5). Anti-CLrP antibody blocking of CLrP reduced the PB of Wt, VC and T. PIS has no significant effect on the PB. AntiGRP78 an irrelevant antibody showed no significant difference in the infectivity of T.

## Discussion

CLrP belongs to the protein superfamily of LRR, which shows a strong selection pressure for the conservation of this motif among several organisms [37, 39]. In eukaryotes, the LRR motif is found in proteins encoded by the disease resistance (R) genes of the plant immune system [40] and by the toll and toll-like genes of Drosophila and mammals, which are implicated in innate immune responses [41,42]. Also internalins of *L*. *monocytogenes* have distinctive leucine-rich repeats (LRR) which play crucial role in interaction with human intestinal mucin MUC2 [43].

The upregulated expression of CLrP has been recently demonstrated in a SAG resistant isolate of *L*. *donovani* (13). Persistent sensitivity profile of clinical isolates employed in this study has been checked earlier through *in vitro* and *in vivo* (golden hamsters) experiments, confirming the patient’s response to SAG [44]. In order to interpret the role of CLrP in resistance, gene was cloned, expressed and the recombinant protein (~71 kDa) was purified to homogeneity, which exhibited very close homology to several *Leishmania spp*. LdCLrP has 13.56% homology with humans that indicates its potential to be explored as drug target. Further, bioinformatic analysis revealed that out of the whole CLrP, N-terminus part of the protein containing 248 amino acid (~27kDa) shows a novel entity, whereas the remaining part of 374 amino acid (~41kDa) exhibited a molecular pattern of LRR motif (Fig E in S1 Text). Western analysis of Ld promastigotes WCL with polyclonal anti-rLdCLrP antibody has depicted 68kDa, 41kDa and 27kDa bands in the soluble fraction of LdS1 which apparently undergoes proteolytic cleavage probably separating the LRR motif of 41kDa from the novel 27kDa motif. A similar profile was observed in the membrane fraction of LdS1, however, an additional prominent band of ~150kDa was also detected therein. The band patterns were not followed in the purified recombinant protein (~71kDa), revealing the protein is not autocatalytic. Earlier in the proteomics study, the protein was detected only at higher molecular weight (~150kDa) in the membrane fraction [13], and no other cleaved part of the protein was distinguished, not even of its actual size ~68kDa, in any of the membrane as well as soluble fraction indicating the membrane entity of the protein somehow playing a crucial role in SAG resistance. Deglycosylation of whole cell lysate of *L*. *donovani* depicts the post translational modification only in the 150kDa entity as a mobility shift of 150kDa band occurred on SDS-PAGE gel wherein no change is observed in the position of any other entity of the protein (68kDa, 41kDa and 27kDa). Localization study of the protein shows the dual existence of the protein in nuclear as well as in the membrane of parasite. The presence of protein in the membrane as well as the presence of ~150kDa band in western analysis reaffirms the identification of protein in the membrane fraction in proteomics studies [13]. Nuclear existence of CLrP was positively probed by Ld60sRL23a and LdTPI confirming its interaction with DNA. LxLL motif of CLrP (299^th^ position), is well known for nuclear receptor cofactor interactions involved in transcriptional regulations [45]. The above evidences pointed out towards the proteolytic cleavage and post translational modification of the protein throughout the cell.

CLrP transcript contour revealed ~2 fold expression of CLrP transcripts in resistant isolates, verifying the differential proteomics finding [13]. As in *Leishmania* the gene expression is rarely regulated at the transcriptional level and *L*. *donovani* strains have been shown to generate extensive aneuploidy [46, 47], the increase in the CLrP transcripts may therefore be attributable to the ploidy phenomenon. Besides the differential expression of proteins one other phenomenon of drug resistance includes gene amplification / duplication, an example of which is well known in cultured mammalian cells for the acquisition of resistance to MTX3 (Methotrexate) [48,49]. *Leishmania* also adapted themselves for resistance to several cytotoxic compounds often via amplifying or deleting a number of specific loci coding for either drug targets or drug transporters [50, 51, 52].

CLrP’s expression profile further depicts the significantly different expression of 150kDa, 68kDa and 41kDa bands among the clinical isolates wherein 2.1 fold increased expression of 150kDa band was observed in resistant isolates as compared to sensitive ones justifying the proteomics results [13]. On the other hand, the 68kDa and 41kDa band were over-expressed by 2.5 and 1.7 fold respectively in the resistant clinical isolates, which were not noticeable in the proteomics results [13]. The 27kDa band also does not reveal any significant difference among the isolates. As the 150 kDa band is the only glycosylated entity of protein, which is differently overexpressed, it may be argued to have some association with the development of resistance. Nevertheless, it will be too early to directly correlate glycosylation pattern with resistance mechanism as there may be several other players in the pathway(s) associated with the development of SAG resistance in *Leishmania* [44, 53, 54].

To check the potential of CLrP to alter the SAG sensitivity profile of parasite, CLrP was over-expressed in WT [33]. Similar to WT, a pattern of several bands was observed in GFP-CLrP overexpressed cells by western blotting using anti-GFP antibodies, evidencing the proteolytic cleavage of CLrP. This ensures the CLrP expression in its original form. Transfectants were further analyzed *in vitro* in macrophage-amastigote model [Sb (V)] as well as in the promastigotes form [Sb (III)] for their SAG sensitivity profile. Our results indicate that overexpression of CLrP as GFP conjugate in WT *Leishmania* promastigotes as well as amastigotes has decreased the SAG sensitivity and their sensitivity pattern towards Sb(III) and Sb(V) were almost parallel. Thus, increasing concentrations of CLrP and parasite modulation towards SAG revealed its potential in SAG resistance.

In *Leishmania*, CLrP being a member of the LRR super—family is ideally placed on the membrane of the parasite to interact with the macrophages, and there is mounting evidence that the parasite uses a variety of ligands to interact with the host such as membrane bound PPG and LPG [55]. Both of them share LRR motif which plays a crucial role in macrophage invasion [56]. Apparently, the parasite has learned to exploit its LRR motif in host interactions. Hence, the protein macrophage interaction consequences were further investigated for its infectivity. Blocking CLrP in S1 and VC lowered its infectivity to more than 2 folds when compared with control (PIS treatment), whereas treatment of T_50_ with anti-CLrP also decreased its infectivity. VC showed a similar pattern of infectivity that of WT, but infectivity of both decreased 1.6 times as compared to T_50_. These results indirectly indicate that overexpression of CLrP is related to the development of resistance. SSG (Sodium Stibogluconate) resistant parasites have been reported to increase the parasitic burden *in vivo* but its direct link to any molecular marker still remains to be investigated [57]. Also, LRR has been reported to provide a scaffold implicated in assisting several pathogens-associated molecular patterns as well as surface receptors, therefore over-expression of the protein may facilitate the interaction of parasites to macrophages and thus its invasion by the parasites.

The results indicate that the CLrP upregulation is associated with SAG resistance, but it may be linked indirectly. There could be several possibilities for induction of resistance due to CLrP overexpression, such as its association or competition with the cascade(s) involved. The results further seek a longitudinal study to trace the routes of the protein journey and to decipher the consequences of post-translational modification at the functional level and its actual association with SAG resistance.

This study further demonstrates the ability of CLrP in increasing the infectivity of parasites which can smoothen the parasite invasion in macrophages and further modulating parasite behavior for SAG. Validation of the present finding depends on the down-regulation of CLrP, but multi-copy of the CLrP dispersed in the genome of *Leishmania* assures the infeasibility of knocking out its gene. Hence, this study exclusively depends on the consequences of upregulation of CLrP. As the Indian subcontinent largely depends on combination chemotherapy (SAG with other drugs), the parasites seem to have learned to modulated itself in resisting these combinations under several laboratory conditions [58]. Understanding of resistance mechanism is therefore essential to strengthen our arsenal of chemotherapeutic strategies.

## Supporting Information

S1 Text Supplementary Table A Sequences of forward and reverse primers (used in the present study). **Supplementary Table B** Percent identity/similarity of CLrP with different sp. of *Leishmania* and *Homo sapiens*. **Figure A** Phylogenetic tree of CLrP calculated using phylogeny.fr (phylogeny.lirmm.fr) with default values of parameters. **Figure B** LRR motif of *L*. *donovani* (CLrP) protein, which contains six repeats that are shown here, along the repeats found in different organisms, including *Trypanosoma brucei* (ESAG8), *C*. *luciliae* (C6K3N8), *H*.*sapiens* (SKP2_HUMAN), *S*. *cerevisiae* (P38285), *A*. *thaliana* (AT1G69545) and *P*. *patens* (moss) (A9SSK0). Proteins are encoded as uniprot identifier. **Figure C** (A) rLdCLrP expression in *E*. *coli*, purification and elution were done at 300mM of imidazole concentration and separation in 12%SDS PAGE. M: Molecular wt. Markers, Lane 1 Whole cell lysate (WCL) of uninduced *E*. *coli* and Lane2: WCL of *E*. *coli* induced at 18°C with 1mM IPTG; Lane3,4: purified rCLrP (B) western blot analysis of *E*. *coli* (pET28a+ CLrP) using anti-rLdCLrP antibody. M: Molecular mass marker; Lane1: WCL before IPTG induction; Lane2, WCL after IPTG (1mM) induction at 18°C; Lane3: Purified protein; **Figure D** Fold expression of Actin (as internal control) in different clinical isolates of *L*.*donovani*. **Figure E** Bioinformatic analysis of CLrP (A) Positioning of LRR motif in CLrP,(B) LdCLrP protein superimposed on template LLR containing human ribonuclease (PDB id: 1z7x), (C)LRR motif and LXXLL motif positioning in CLrP.
(DOCX)
