# Supplementary material for: Over-Expression of Cysteine Leucine Rich Protein Is Related to SAG Resistance in Clinical Isolates of Leishmania donovani
Source: PLoS Negl Trop Dis. 2015 Aug 21;9(8):e0003992. doi: 10.1371/journal.pntd.0003992 (PMC4546639; doi:10.1371/journal.pntd.0003992)
Supplement: S1 Text — Supplementary Table B Percent identity/similarity of CLrP with different sp. of Leishmania and Homo sapiens. Figure A Phylogenetic tree of CLrP calculated using phylogeny.fr (phylogeny.lirmm.fr) with default values of parameters. Figure B LRR motif of L. donovani (CLrP) protein, which contains six repeats that are shown here, along the repeats found in different organisms, including Trypanosoma brucei (ESAG8), C. luciliae (C6K3N8), H.sapiens (SKP2_HUMAN), S. cerevisiae (P38285), A. thaliana (AT1G69545) and P. patens (moss) (A9SSK0). Proteins are encoded as uniprot identifier. Figure C (A) rLdCLrP expression in E. coli, purification and elution were done at 300mM of imidazole concentration and separation in 12%SDS PAGE. M: Molecular wt. Markers, Lane 1 Whole cell lysate (WCL) of uninduced E. coli and Lane2: WCL of E. coli induced at 18°C with 1mM IPTG; Lane3,4: purified rCLrP (B) western blot analysis of E. coli (pET28a+ CLrP) using anti-rLdCLrP antibody. M: Molecular mass marker; Lane1: WCL before IPTG induction; Lane2, WCL after IPTG (1mM) induction at 18°C; Lane3: Purified protein; Figure D Fold expression of Actin (as internal control) in different clinical isolates of L.donovani. Figure E Bioinformatic analysis of CLrP (A) Positioning of LRR motif in CLrP,(B) LdCLrP protein superimposed on template LLR containing human ribonuclease (PDB id: 1z7x), (C)LRR motif and LXXLL motif positioning in CLrP. (DOCX) [file pntd.0003992.s001.docx]

**S1 TEXT (SUPPLEMENTARY MATERIAL)**

**Table A** : Sequences of forward and reverse primers ( used in the present study).

| **S.No.** | **Primer** | **Sequences** | **Amplification For** |
| --- | --- | --- | --- |
| 1 | Forward | 5’ GGATCCATGGCTGACCCTGCGCACACCC 3’ | Cloning in pTZ57R/T (T/A) & pET28a(+) |
| 2. | Reverse | 5’ GAATTCTTACGGAAAGGTGTGTGCCTCAGCTG 3’ |  |
| 3 | Forward | 5’ GGATCCATGGCTGACCCTGCGCACACCC 3’ | Cloning in pXG-‘GFP+ |
| 4. | Reverse | 5’GATATCCGGAAAGGTGTGTGCCTCAGCTGC 3’ |  |
| 6. | Forward | 5’ATGCATATCACCTCGCTAAA 3’ | Real Time (CLrP) |
| 7. | Reverse | 5’ CTTCAAGGTGAATGACAGAT 3’ |  |
| 8. | Forward | 5’ CAACATTGTGCTGTCCGGTG 3’ | Real Time (Actin) |
| 9. | Reverse | 5’ CCGATCCACACGCTGTACTT 3’ |  |
| 10. | Forward | 5’GGTACCATGCCTCCTGCTCAGAAG3’ | 60s Ribosomal L23a |
| 11. | Reverse | 5’ AAGCTTGACAAGACCGATCTT 3’ |  |
| 12. | Forward | 5’GGATCCATGTCCGCCAAGCCCCAGCCGATC 3‘ | LdTPI (Triose Phosphate Isomerase, ) |
| 13. | Reverse | 5’ GAATTCTCACTTCCGGGTGGCATCAATGATGTC 3’ |  |

**TABLE B**: Percent identity of CLrP with different sp. of *Leishmania* and *Homo sapiens.*

| **S.No.** | **Species** | **Identity** |
| --- | --- | --- |
| 1 | *L.infantum* | 99.19 |
| 2. | *L. major* | 95.18 |
| 3. | *L.maxicana* | 92.04 |
| 4 | *L.brazilliensis* | 82.14 |
| 5 | *L.tarentole* | 81.12 |
| 6. | *Crithidia Sp.* | 66.19 |
| 7. | *H.sapiens* | 13.56 |
| 8. | *T.vivax* | 7.96 |


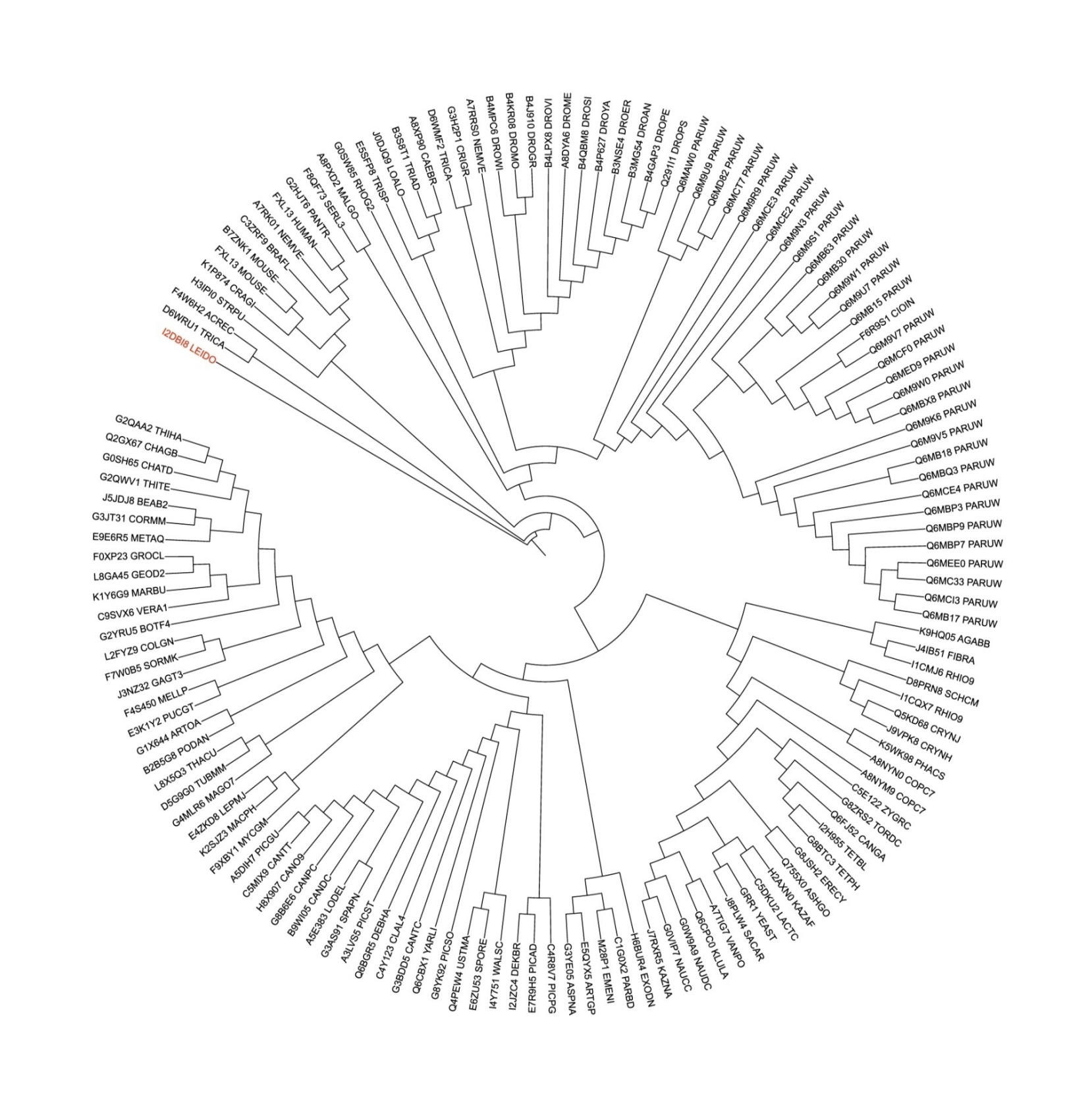


**FIG. A**. Phylogenetic tree of CLrP calculated using phylogeny.fr (phylogeny.lirmm.fr) with default values of parameters.


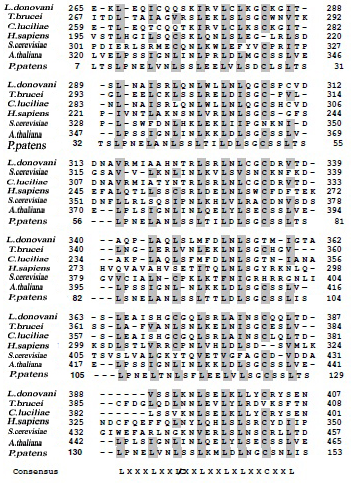


**FIG. B**. LRR motif of *L. donovani* (CLrP) protein which contains six repeats that are shown here along the repeats found in different organisms including *Trypanosoma brucei* (ESAG8), *C.luciliae* (C6K3N8) , *H.sapiens* (SKP2_HUMAN), *S.cerevisiae* (P38285), *A.thaliana* (AT1G69545) and ***P.*** *patens* (moss) (**A9SSK0**). Proteins are encoded as uniprot identifier.

**
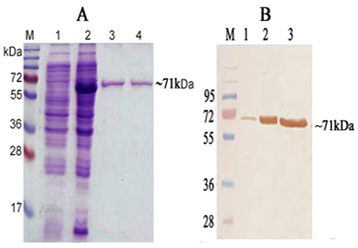
**

**Fig. C** (A) rLdCLrP expression in *E. coli*, purification and elution were done at 300mM of imidazole concentration and separation in 12%SDS PAGE. M: Molecular wt. Markers, Lane 1 Whole cell lysate (WCL) of uninduced *E. coli* and Lane2: WCL of *E. coli* induced at 18°C with 1mM IPTG; Lane3,4: purified rCLrP (B) western blot analysis of *E. coli* (pET28a+ CLrP) using anti-rLdCLrP antibody. M: Molecular mass marker; Lane1: WCL before IPTG induction; Lane2, WCL after IPTG (1mM) induction at 18°C; Lane3: Purified protein;

**
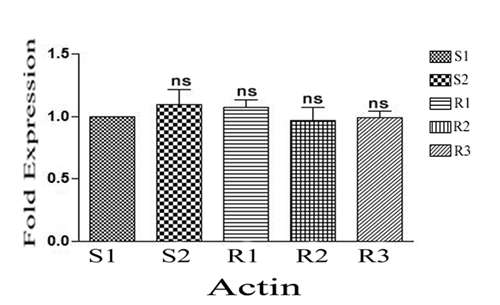
**

**Fig. D.** Fold expression of Actin (as internal control) in different clinical isolates of *L.donovani.*

**
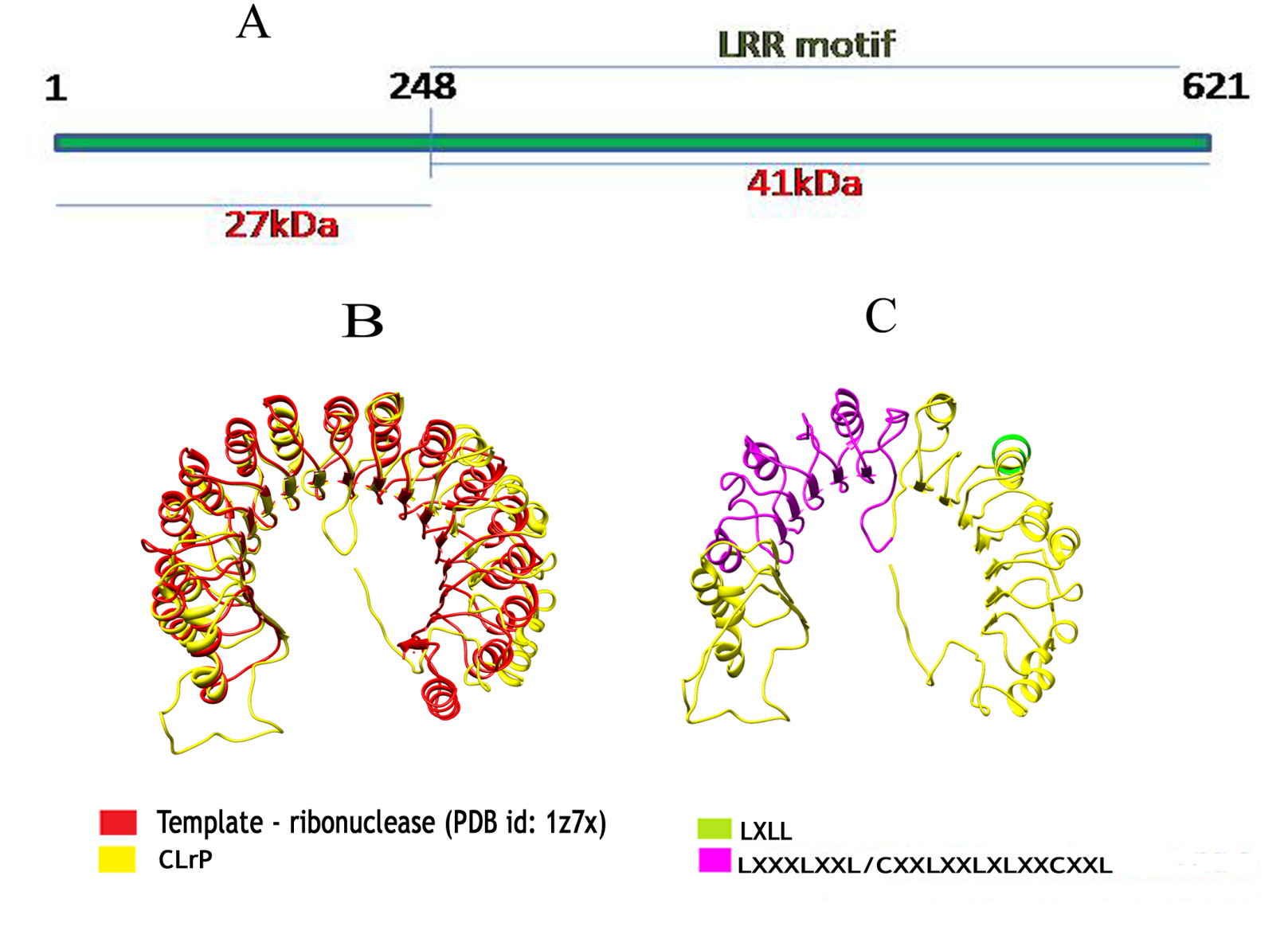
**

**FIG.E**. **Bioinformatic analysis of CLrP** (A) Positioning of LRR motif in CLrP,(B) LdCLrP protein superimposed on template LLR containing human ribonuclease (PDB id: 1z7x), (C)LRR motif and LXXLL motif positioning in CLrP.
